# Hemodynamic effects of red blood cell transfusion in patients with hemato-oncologic diseases

**DOI:** 10.3389/fmed.2026.1711829

**Published:** 2026-02-19

**Authors:** Babak Yazdani, Nikoletta Schneider, Mohammed Abba, Mark Schulz, Gerhard Schumacher, Goekhan Yuecel, Ralf-Dieter Hofheinz, Daniel Duerschmied, Sabine Kayser, Anna Hohneck

**Affiliations:** 1 Fifth Department of Medicine (Nephrology, Hypertensiology, Rheumatology, Endocrinology, Diabetology, Pneumology), Faculty of Medicine of the University of Heidelberg, University Medical Center Mannheim UMM, Mannheim, Germany; 2 Department of Hematology and Oncology, Medical Faculty Mannheim, University Medical Centre Mannheim, Heidelberg University, Mannheim, Germany; 3 Harzklinikum Dorothea Christiane Erxleben - Klinikum Wernigerode, Klinik für Diagnostische und Interventionelle Radiologie, Wernigerode, Germany; 4 inmediQ GmbH, Butzbach, Germany; 5 Department of Cardiology, Haemostaseology, and Medical Intensive Care, Medical Faculty Mannheim, University Medical Centre Mannheim, Heidelberg University, Mannheim, Germany; 6 European Center for AngioScience (ECAS), German Centre for Cardiovascular Research (DZHK) partner site Heidelberg/Mannheim, and Centre for Cardiovascular Acute Medicine Mannheim (ZKAM), Mannheim, Germany; 7 Medical Faculty Mannheim, German Red Cross Blood Service Baden-Württemberg-Hessen, Institute of Transfusion Medicine and Immunology, Heidelberg University, Mannheim, Germany; 8 Department of Endocrinology, Diabetology, Metabolism and Clinical Chemistry, Heidelberg University Hospital and German Center for Diabetes Research (DZD) associated partner site Heidelberg, Heidelberg, Germany

**Keywords:** anemia, augmentation index, blood transfusion, central and peripheral hemodynamics, left ventricular ejection time

## Abstract

**Introduction:**

The hemodynamic effects of red blood cell (RBC) transfusion in patients with hemato-oncologic diseases can be adverse. We measured acute hemodynamic changes with a novel non-invasive monitoring device.

**Methods:**

Twenty-six patients (9 female, 17 male; median age 73) with hematological malignancies or solid tumors were included. Peripheral and central hemodynamics were assessed before and after RBC transfusion using the VascAssist2.0 device.

**Results:**

Baseline hemoglobin was 7.8 g/dL (range 6.2–8.7). Post-transfusion, significant hemodynamic changes were observed: heart rate decreased from 81 to 74 bpm (*p* < 0.0001). Both brachial and aortic systolic and diastolic blood pressures increased significantly (brachial SBP: 123–128 mmHg, *p* = 0.001; aortic SBP: 101–109 mmHg, *p* = 0.0003; brachial DBP: 62–64 mmHg, *p* = 0.01; aortic DBP: 61–64 mmHg, *p* = 0.02). The augmentation index adjusted to 75 bpm (AIx75) also rose significantly (11–18%, *p* = 0.003). There were non-significant trends toward decreased aortic pulse wave velocity (*p* = 0.09) and increased estimated blood viscosity (*p* = 0.053), indicating possible beneficial rheological effects.

**Conclusion:**

RBC transfusion in patients with hemato-oncologic diseases resulted in significant increases in central and peripheral blood pressures and a reduction in heart rate in our pilot study. Trends toward improved blood rheology and vascular function were observed, though not statistically significant. While these immediate hemodynamic effects appear beneficial, further research is needed to determine long-term outcomes, especially for patients requiring frequent transfusions.

## Graphical abstract

**Figure:**
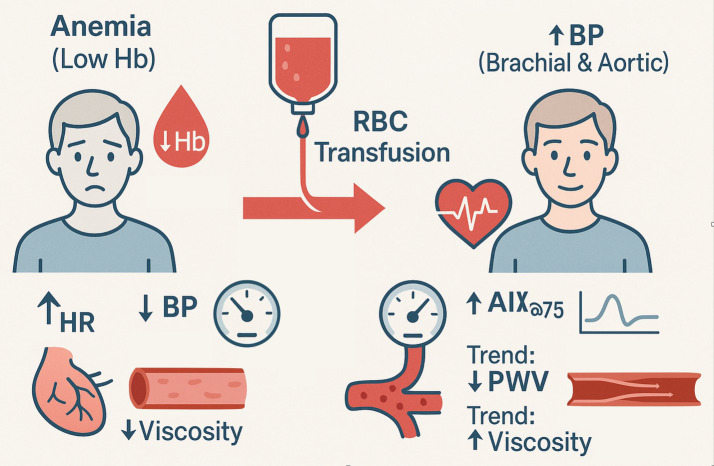

## Introduction

Oxygen is primarily delivered to organs and tissues bound to hemoglobin within red blood cells (RBCs). Consequently, anemia may impair oxygen delivery and lead to end-organ undersupply. However, in most patients, compensatory mechanisms such as increased cardiac output and enhanced oxygen extraction from RBCs help maintain adequate oxygen supply across a wide range of hemoglobin levels (1). Oxygen delivery (DO₂) is estimated by the equation: DO₂ = cardiac output × arterial oxygen content. While healthy individuals can typically augment DO₂ by increasing cardiac output through elevated heart rate or stroke volume, critically ill patients often rely more on arterial oxygen content. In healthy, unmedicated individuals, it has been shown that heart rate increases linearly with decreasing hemoglobin levels during acute isovolemic anemia (2). In line with this, the administration of two RBC units can reduce heart rate by more than 10 bpm (3).

However, the hemodynamic response to anemia and transfusion may differ in patients with hemato-oncologic diseases. Therefore, blood transfusion remains a cornerstone therapy for managing anemia in patients with hemato-oncologic diseases, yet its hemodynamic consequences reveal a complex interplay between erythrocyte biology, circulatory physiology, and evolving monitoring technologies (4, 5). Importantly, transfusion thresholds have shifted toward restriction (7.0–8.0 g/dL), reducing RBC exposure by up to 41% in diverse patient populations, without adversely affecting 30-day mortality or major morbidity versus liberal strategies (5, 6). Patient selection is crucial, as most recent transfusion studies enrolled patients with substantial comorbidity, acute illness, or perioperative risk factors. The evidence base, however, is not unequivocal: While the TRICC trial demonstrated reduced in-hospital mortality for restrictive transfusion (22.3% vs. 28.1%, *p* = 0.05), other studies observed divergent outcomes (6). The TITRe2 trial in cardiac surgery found higher mortality in the restrictive group (*p* = 0.045), highlighting cohort-dependent heterogeneity (7). Furthermore, Villanueva et al. showed restrictive transfusion in acute GI bleeding led to improved clinical outcomes compared to liberal strategies (8). The complex relationship between anemia, transfusion, and patient outcomes is further supported by evidence that preoperative anemia predisposes to higher 30-day morbidity and mortality across surgical cohorts (9). Transfusion appropriateness remains variable in clinical practice. Recent monocentric data by Merolle et al. (10) (*n* = 600 cancer patients) illustrate that implementation of a two-step patient blood management protocol increased appropriate RBC transfusions from 38 to 79%, while reducing average units transfused per patient by 1.3. The authors concluded, that a structured PBM program specifically dedicated to surgical oncology should cover the entire perioperative period and might further improve transfusion appropriateness in these patients.

Additional sources of transfusion risk include prolonged storage of RBCs, which leads to “storage lesions”—biochemical and biomechanical changes that compromise cell function and survival, potentially impairing oxygen delivery and increasing morbidity and mortality (4). Our prior work found that blood donation in healthy subjects acutely decreased systolic blood pressure, augmentation index (AIx), and left ventricular ejection time (LVET), indicating reversible changes in left ventricular function and arterial wave reflections, although central blood pressure remained stable (11).

Against this background, we sought to investigate the hemodynamic consequences of RBC transfusion—particularly on central and peripheral parameters—using the VascAssist 2.0 device. This non-invasive system utilizes radial pulse wave analysis (PWA) based on the Westerhof multicompartment model, providing metrics of arterial stiffness (pulse wave velocity, PWV), vascular resistance and compliance, AIx, and LVET (12). Central blood pressure measurements with VascAssist 2.0 are comparable to gold-standard devices (SphygmoCor), with advantages of minimal operator dependency and applicability in arrhythmic patients (13).

## Materials and methods

This prospective, single-center study was conducted at the University Medical Center Mannheim, Medical Faculty Mannheim, Heidelberg University, Germany, between May and August 2024. The study was approved by the local ethical committee of the Medical Ethics Commission II, Faculty of Medicine Mannheim, University of Heidelberg, Germany (2021-410 M-§ 47 (3) MPDG). Written informed consent was obtained from all patients prior to study inclusion. The study was conducted in accordance with the Declaration of Helsinki, and data protection complied with the EU Data Protection Directive.

Eligibility criteria included male and female patients aged 18 years or older with a diagnosis of a hematologic malignancy or solid tumor and clinical indication for RBC transfusion and the ability to provide informed consent (Table 1). Exclusion criteria were hemodynamic instability, ongoing acute bleeding, pregnancy and inability to comply with study procedures.

**Table 1:** Baseline characteristics.

Baseline characteristics	*N* = 26
Sex, female (%)	9 (34.6)
Age, years	73 (range, 42–84)
Height, cm	171 (156–188)
Weight, kg	70 (50–95)
BMI, kg/m^2^	22.7 (22.1; 25.6)
SBP, mmHg	147 (134; 163)
DBP, mmHg	83 (70; 90)
Heart rate, bpm	81 (70; 87)
Hb, g/dl	7.8 (6.2–8.7)
Platelets, 10^9^/l	189 (100; 294)
eGFR, ml/min/1.73 m^2^	57 (36; 86)
Creatinine, mg/dl	1.1 (0.8; 1.7)
Concomitant cardiovascular diseases, *n* (%)	
CAD	6 (23.1)
PAD	2 (7.7)
Atrial fibrillation	5 (19.2)
Arterial hypertension	14 (53.8)
Diabetes mellitus	2 (7.7)
Concomitant medications of special interest (CMSI), *n* (%)	
Betablocker	11 (42.3)
ARB	8 (30.8)
CCB	3 (11.5)
Diuretics	9 (34.6)
Statin	5 (19.2)
ASA	7 (26.9)
Anticoagulants	10 (38.5)
Underlying hemato-oncologic disease, *n* (%)	
Solid tumors (ovarian cancer, colorectal cancer, urothelial cancer, thymoma, NSCLC)	9 (34.6)
Leukemias (AML, CLL)	5 (19.2)
Myelodysplastic syndromes	6 (23.1)
Myeloproliferative neoplasia	3 (11.5)
Plasma cell neoplasm	2 (7.7)
B-NHL	1 (3.8)

Data are presented as median (range/IQR) or frequencies.

AML, acute myeloid leukemia; ASA, Acetylsalicylic acid; ARB, Angiotensin receptor blocker; BMI, body-mass-index; B-NHL: B-cell non-Hodgkin lymphoma; CLL, chronic lymphocytic leukemia; SBP, systolic blood pressure; DBP, diastolic blood pressure; bpm, beats per minute; CAD, coronary artery disease; CCB, calcium channel blocker; eGFR, estimated glomerular filtration rate; Hb, hemoglobin; NSCLC, non-small cell lung cancer; PAD, peripheral artery disease.

Transfusion was performed with whole blood units rather than packed red blood cells. Each unit had a standard volume of approximately 250–300 mL, and most patients received 1–2 units, according to clinical indication and local transfusion protocols. Chronic cardiovascular medications were continued at unchanged doses during the study, and no new vasoactive drugs were introduced between pre- and post-transfusion measurements.

Peripheral and central hemodynamics were assessed using non-invasive oscillometric measurements with the VascAssist 2 device (inmediQ GmbH, Butzbach, Germany), after resting in supine position for >10 min. Before and after RBC transfusion. Brachial and radial cuffs were placed on both arms. After calibration and internal testing of all cuffs, pulse waves were recorded three times with a 30-s interval between each measurement. The recorded pulse waves were analyzed offline using proprietary software.

Central blood pressure estimates, including central PWV and AIx, were validated against invasive measurements and non-invasive applanation tonometric pulse wave analysis (SphygmoCor, AtCor Medical, Sydney, Australia), as previously described. The central PWV was determined by measuring the time difference between the first and second peaks of the brachial pulse wave, with the second peak resulting from the reflection of the peripheral pulse wave. AIx, defined as the increase in blood pressure due to pulse wave reflection, was obtained from the calculated aortic pulse curve and adjusted to a heart rate of 75 beats per minute (AIx@75). In addition to standard hemodynamic parameters, the VascAssist 2.0 software calculates an index of whole blood viscosity (*estimated blood viscosity*) based on a viscoelastic vascular model fitted to the recorded oscillometric pulse waves. This algorithm uses the shape and timing characteristics of the brachial and radial pulse waveforms to estimate vascular resistance and estimated blood viscosity, which correlates with whole blood viscosity at low shear rates. This method has been validated in previous work comparing oscillometric pulse wave–derived mechanical parameters with established reference techniques for vascular stiffness and central hemodynamics and demonstrates high measurement accuracy and requires minimal operator training and patient cooperation (14).

### Statistics

Statistical analysis was performed using GraphPad Prism version 10.4.1 (GraphPad Software Inc., La Jolla, California, United States). Continuous variables are presented as median (interquartile range, IQR) unless otherwise stated. Pre- and post-transfusion values were compared using the Wilcoxon signed-rank test for paired samples. A two-sided *p*-value < 0.05 was considered statistically significant.

## Results

### Baseline characteristics

A total of 26 patients were included in the study. The median age was 73 years (range: 42–84), and 34.6% were female. The median height and weight were 171 cm (IQR: 156–188) and 70 kg (IQR: 50–95), respectively, resulting in a median BMI of 22.7 kg/m^2^ (IQR: 22.1–25.6). Median systolic and diastolic blood pressures were 147 mmHg (IQR: 134–163) and 83 mmHg (IQR: 70–90), respectively, and the median heart rate was 81 bpm (IQR: 70–87). The median hemoglobin level was 7.8 g/dL (range: 6.2–8.7) and median platelet counts were 189 × 10^9^/l (IQR: 100; 294). Renal function was moderately impaired, with a median eGFR of 57 mL/min/1.73 m^2^ (IQR: 36–86) and median serum creatinine of 1.1 mg/dL (range: 0.8–1.7). Cardiovascular comorbidities were common: 23.1% had coronary artery disease (CAD), 7.7% peripheral artery disease (PAD), 19.2% atrial fibrillation, and 53.8% arterial hypertension. Diabetes mellitus was present in 7.7% of patients. Concomitant medications included betablockers (42.3%), angiotensin II receptor blockers (ARBs, 30.8%), calcium channel blockers (CCBs, 11.5%), diuretics (34.6%), statins (19.2%), acetylsalicylic acid (ASA, 26.9%), and anticoagulants (38.5%).

Regarding the underlying hematologic or oncologic diseases, 34.6% of patients had solid tumors, followed by myelodysplastic syndromes (23.1%), leukemias (19.2%), myeloproliferative neoplasia (11.5%), plasma cell neoplasms (7.7%) and lymphoma (3.8%) (Table 1).

### Central and peripheral hemodynamic parameters before and after red blood cell transfusion

**Figure 1:**
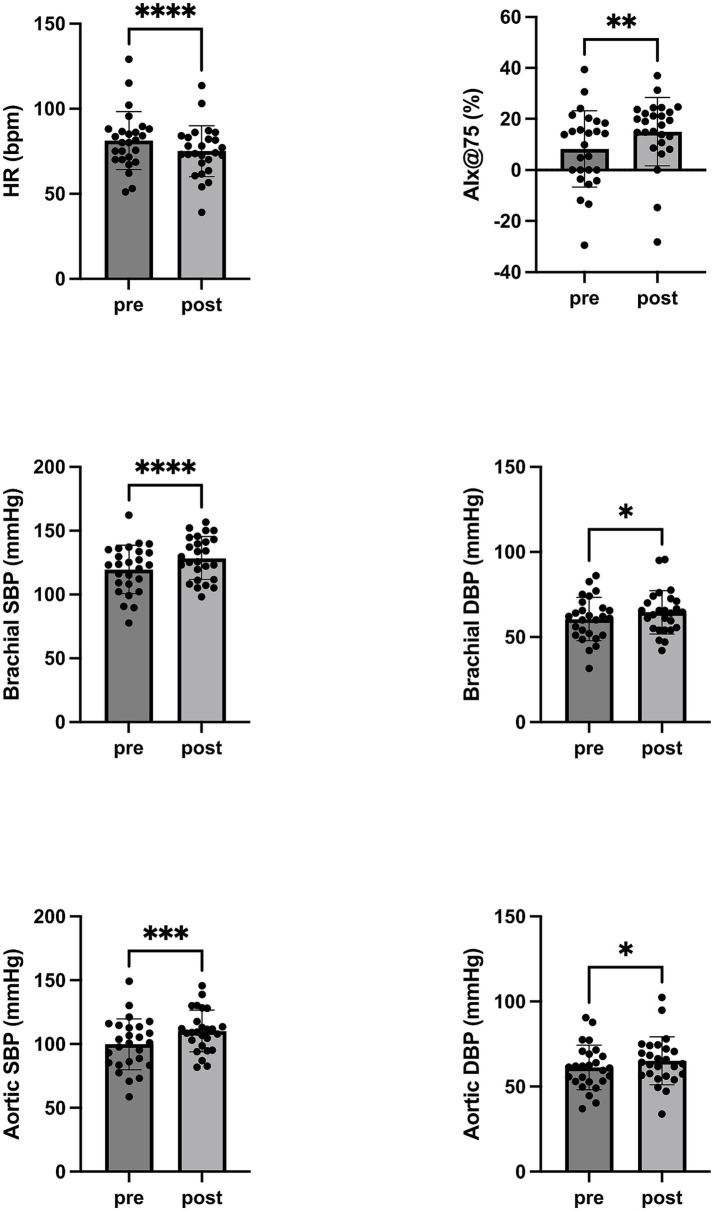
Central and peripheral hemodynamic parameters before and after red blood cell transfusion. Changes in central and peripheral hemodynamic parameters before and after red blood cell transfusion. Median, interquartile range, and outliers for heart rate (HR), systolic (SBP), and diastolic blood pressure (DBP) and augmentation index (AIx@75) are shown. The paired values were compared using the Wilcoxon test; *p* values are indicated as follows: **** < 0.0001, *** < 0.001, ** < 0.01, * < 0.05.

Following RBC transfusion, significant changes in hemodynamic parameters were observed (Table 2). Heart rate decreased from median 81 (IQR: 71–87) to median 74 (IQR: 66–83) beats per minute (*p* < 0.0001). Both brachial systolic and diastolic blood pressures increased significantly, from 123 (IQR: 109–133) to 128 (IQR: 116–142) mmHg (*p* = 0.0001) and from 62 (IQR: 52–66) to 64 (IQR: 55–72) mmHg (*p* = 0.01), respectively. Similarly, central aortic systolic and diastolic blood pressures increased from median 101 (IQR: 86–113) to median 109 (IQR: 101–116) mmHg (*p* = 0.0003) and from median 61 (IQR: 53–68) to median 64 (IQR: 57–73) mmHg (*p* = 0.02), respectively. The augmentation index adjusted to a heart rate of 75 bpm (AIx@75) increased from 11% (IQR: 0–15) to 18% (IQR: 12–22) (*p* = 0.003). Although not reaching statistical significance, several parameters showed noteworthy trends. Central pulse wave velocity (PWV) decreased from median 9.6 (IQR: 8.2–10.7) to median 8.4 (IQR: 7.6–9.8) m/s (*p* = 0.09). Both vascular stiffness and peripheral resistance increased numerically from median 72 (IQR: 39–91) % to median 85 (IQR: 47–102) % (*p* = 0.052) and from median 58 (IQR: 36–71) % to median 70 (IQR: 51–85) % (*p* = 0.08), respectively, and were in trend statistically significant. Similarly, estimated blood viscosity rose from median 3.2 (IQR: 2.0–4.2) to median 4.0 (IQR: 2.8–5.1) mPa·s (*p* = 0.053), reflecting a trend toward increased hemorheological load (Figure 1).

**Table 2:** Central and peripheral hemodynamic parameters.

Hemodynamic parameters	Pre	Post	*p-*value
Heart rate, bpm	81 (71; 87)	74 (66; 83)	**<0.0001**
Brachial SBP, mmHg	123 (109; 133)	128 (116; 142)	**0.0001**
Brachial DBP, mmHg	62 (52; 66)	64 (55; 72)	**0.01**
Central PWV, m/s	9.6 (8.2; 10.7)	8.4 (7.6; 9.8)	0.09
Stiffness, %	72 (39; 91)	85 (47; 102)	0.052
Resistance, %	58 (36; 71)	70 (51; 85)	0.08
Estimated blood viscosity, mPa·s	3.2 (2.0; 4.2)	4.0 (2.8; 5.1)	0.053
Aortic SBP, mmHg	101 (86; 113)	109 (101; 116)	**0.0003**
Aortic DBP, mmHg	61 (53; 68)	64 (57; 73)	**0.02**
AIx75, %	11 (0; 15)	18 (12; 22)	**0.003**
LVET, ms	260 (225; 271)	253 (227; 272)	0.50

Data are presented as median (IQR). Bold values mark statistical significance.

Aix75, augmentation index adjusted to a heart rate of 75 beats per minute; SBP, systolic blood pressure; DBP, diastolic blood pressure; bpm, beats per minute; LVET, left ventricular ejection time; PWV, pulse wave velocity.

When stratified by underlying disease, hematological and oncological patients showed largely comparable hemodynamic responses to RBC transfusion. In both subgroups, transfusion led to a significant reduction in heart rate and an increase in both brachial and aortic systolic blood pressure. The augmentation index (AIx@75) rose significantly in each group, whereas left ventricular ejection time remained unchanged. Arterial stiffness and resistance increased numerically after transfusion in both subgroups without reaching statistical significance, and central PWV declined slightly in both groups, again without a significant between-group difference. Importantly, analysis of transfusion-induced changes (*Δ* post–pre) did not identify any significant differences between the two subgroups for any hemodynamic parameter, indicating a similar vascular response to transfusion irrespective of the underlying disease (Supplementary Table S1).

## Discussion

Our results provide a comprehensive overview of the physiological changes that occur following RBC transfusion in patients with anemia due to hematologic or oncologic disease. We observed that RBC transfusion in this vulnerable, predominantly elderly population with a high prevalence of cardiovascular comorbidities led to significant hemodynamic changes, in line with previous examinations (15, 16). The most notable finding was a significant reduction in heart rate after transfusion, which likely reflects a compensatory response to improved oxygen-carrying capacity and reduced sympathetic drive. This is consistent with previous studies demonstrating that RBC transfusion can attenuate the tachycardic response to anemia (17, 18). In an exploratory subgroup analysis, patients with hematological and oncological disease exhibited broadly similar transfusion-related changes in central and peripheral hemodynamics, suggesting that the acute vascular response to RBC transfusion is largely independent of the underlying etiology of anemia.

Both peripheral and central blood pressures increased significantly following transfusion. These increases are physiologically plausible and may be attributed to the combined effects of increased circulating blood volume and higher estimated blood viscosity, which together enhance cardiac preload and systemic vascular resistance (19). The observed rise in augmentation index (AIx@75) further supports the notion of increased wave reflection and arterial tone, potentially due to augmented circulating volume and vascular reactivity (20).

Although some hemodynamic parameters did not reach statistical significance, they showed clear numerical trends. For example, central pulse wave velocity (PWV) tended to decrease, suggesting improved central vascular compliance, while vascular stiffness and peripheral resistance tended to increase. This apparent discrepancy may reflect differential vascular responses: elastic central arteries may become more distensible with volume expansion, whereas peripheral muscular arteries may respond with compensatory vasoconstriction, possibly due to increased blood viscosity or neurohumoral activation (21, 22).

Our findings are in line with the physiological pathways that anemia reduces blood viscosity and vascular resistance, leading to compensatory up-regulation of heart rate and cardiac output (23). RBC transfusion reverses these changes, increasing viscosity and resistance, and normalizing heart rate and blood pressure. The trends toward improved central compliance and increased peripheral resistance highlight the complex interplay of vascular adaptation following transfusion (17, 19). Indeed, estimated blood viscosity increased and slightly missed level of significance, possibly due to the small sample size. This trend reflects the expected hemorheological consequence of an increased erythrocyte mass. Although this parameter was only trendwise statistically significant, the trend underscores the potential trade-off between improved oxygen-carrying capacity and increased vascular workload post-transfusion.

Limitations of our study include the small sample size, single-center design, and lack of long-term follow-up, which are a consequence of the pilot character of our study. Furthermore, the study population was heterogeneous with respect to underlying disease and comorbidities, which may limit generalizability. Future studies with larger cohorts and longitudinal follow-up are warranted to confirm these findings and explore the long-term clinical impact of transfusion-related hemodynamic changes, particularly in patients requiring repeated transfusions. Concomitant cardiovascular medications such as beta-blockers, renin–angiotensin system blockers, calcium channel blockers, diuretics, and antiplatelet or anticoagulant agents may modulate baseline hemodynamics and vascular tone and cannot be fully disentangled from transfusion effects, even though all chronic therapies were continued at unchanged doses during the short observation period. The use of whole blood rather than leukoreduced packed RBC may have influenced volume load, plasma composition and rheology, and should therefore be considered when interpreting our findings. However, this transfusion strategy reflects routine practice at our institution during the study period, and we chose to report it transparently and to acknowledge product type as a potential limitation. Future studies using standardized leukoreduced packed RBC units could help to further delineate the impact of red cell product characteristics on hemodynamic and hemorheological responses. A further limitation of our study is the lack of systematic documentation regarding the underlying cause of anemia. Although anemia in patients with malignancy is often multifactorial, its primary mechanism can vary substantially–ranging from direct marrow infiltration or suppression (e.g., in leukemia or myelodysplastic syndromes) to treatment-related effects (such as chemotherapy-induced cytopenia) or chronic blood loss associated with tumor burden (e.g., gastrointestinal malignancies). Importantly, patients with hemodynamic instability or acute bleeding at the time of transfusion were excluded from this analysis to ensure a more homogeneous study population and to minimize confounding by acute volume loss. Nevertheless, these differing pathophysiologic mechanisms may influence baseline hemodynamic status as well as the cardiovascular response to transfusion. Moreover, our study did not systematically investigate the independent contribution of platelet count or platelet function to hemodynamic and viscosity changes. Future studies with larger sample sizes and concomitant laboratory measurements of whole blood and plasma viscosity, ideally combined with detailed platelet phenotyping, will be needed to clarify the rheological role of platelets in this context. Future prospective studies should aim to better characterize the etiology of anemia in this patient population to enable stratified analyses and a more nuanced understanding of transfusion-related hemodynamic effects.

In conclusion, RBC transfusion in patients with hemato-oncologic diseases induces significant alterations in both central and peripheral hemodynamics, characterized by increased central and brachial SBP, DBP and a reduction in heart rate. The observed tendencies toward lower pulse wave velocity and higher estimated blood viscosity may indicate transient improvements in hemorheology and vascular function. Although these acute effects appear largely favorable, further research is warranted to elucidate the long-term impact, especially in patients undergoing repeated transfusions.

## Data Availability

The raw data supporting the conclusions of this article will be made available by the authors, without undue reservation.
