# Supplementary material for: Hemodynamic effects of red blood cell transfusion in patients with hemato-oncologic diseases
Source: Front Med (Lausanne). 2026 Feb 19;13:1711829. doi: 10.3389/fmed.2026.1711829 (PMC12960126; doi:10.3389/fmed.2026.1711829)
Supplement: Supplementary file 1 [file Table_1.docx]

**Supplementary Table 1. Change in central and peripheral hemodynamic parameters after red blood cell transfusion in hematologic vs oncologic patients**

| **Hemodynamic parameter** | **Hematologic patients (n = 15) Δ post–pre** | **Oncologic patients (n = 11) Δ post–pre** | **p-value (between groups)** |
| --- | --- | --- | --- |
| Heart rate, bpm | -7 ( -13 to -3 ) | -6 ( -11 to -2 ) | n.s. |
| Brachial SBP, mmHg | +6 ( +2 to +15 ) | +7 ( +3 to +15 ) | n.s. |
| Brachial DBP, mmHg | +3 ( 0 to +10 ) | +4 ( 0 to +9 ) | n.s. |
| Aortic SBP, mmHg | +7 ( +3 to +14 ) | +8 ( +4 to +13 ) | n.s. |
| Aortic DBP, mmHg | +3 ( 0 to +9 ) | +3 ( 0 to +7 ) | n.s. |
| AIx@75, % | +7 ( +3 to +10 ) | +6 ( +3 to +11 ) | n.s. |
| Central PWV, m/s | -1.0 ( -1.8 to 0 ) | -0.9 ( -1.6 to 0 ) | n.s. |
| Stiffness, % | +12 ( +3 to +24 ) | +11 ( +2 to +23 ) | n.s. |
| Resistance, % | +12 ( +3 to +23 ) | +11 ( +2 to +22 ) | n.s. |
| Estimated blood viscosity, mPa·s | +0.8 ( +0.3 to +1.0 ) | +0.7 ( +0.3 to +0.9 ) | n.s. |
| LVET, ms | -5 ( -20 to +5 ) | -4 ( -18 to +6 ) | n.s. |

Data are presented as median (IQR) of the absolute change (post–pre). P-values refer to between-group comparisons (hematologic vs oncologic), Wilcoxon rank-sum test.
n.s., not significant; AIx@75, augmentation index adjusted to 75 bpm; SBP, systolic blood pressure; DBP, diastolic blood pressure; PWV, pulse wave velocity; LVET, left ventricular ejection time.
